# Seafood not from the sea: examining consumer behavioral intentions toward plant-based seafood

**DOI:** 10.3389/fnut.2026.1782036

**Published:** 2026-03-10

**Authors:** Min-Yen Chang, Ching-Tzu Chao, Jia-Hong Chen, Han-Shen Chen

**Affiliations:** 1 Department of Accounting, Jiaxing University, Jiaxing, China; 2 Department of Health Industry Technology Management, Chung Shan Medical University, Taichung, Taiwan; 3 Department of Medical Management, Chung Shan Medical University Hospital, Taichung, Taiwan

**Keywords:** consumer behavior, food neophobia, plant-based seafood, sustainable diet, technological trust

## Abstract

The intensifying challenges of climate change and marine resource depletion have propelled plant-based seafood to become the forefront of sustainable food innovations. Although this sector has experienced remarkable technological advancements globally, empirical research on consumer acceptance, particularly in Taiwan, remains limited. This study examines consumer behavioral intentions toward plant-based seafood by integrating value-belief-norm (VBN) theory with the dimensions of food neophobia and technological trust. Through structural equation modeling analysis of data from 280 Taiwanese consumers, our study yielded three significant findings. First, biospheric values strongly influence environmental beliefs (*β* = 0.913, *p* < 0.001), which subsequently shape personal moral norms and purchase and advocacy intention. Second, environmental beliefs function as critical mediators between biospheric values and moral norms, underscoring the essential role of environmental consciousness in consumer decision making. Third, technological trust directly predicts purchase and advocacy intention (*β* = 0.528, *p* < 0.001). These findings contribute to both theoretical understanding and practical applications in sustainable food adoption. We recommend that industry stakeholders enhance product transparency through certification systems and emphasize environmental benefits to foster consumer acceptance. This research advances our understanding of the psychological mechanisms driving sustainable food choices, while providing actionable insights for market development.

## 1 Introduction

In recent years, issues related to climate change and excessive greenhouse-gas emissions have become significant global challenges. The Intergovernmental Panel on Climate Change (IPCC) (1) has emphasized that global warming and intensive agricultural and fishing activities jointly contribute to the degradation of ecosystems, posing serious threats to environmental sustainability. Zhang et al. (2) identified the marine fishing industry as the sector with the highest carbon dioxide emissions. The Food and Agriculture Organization (FAO) of the United Nations (2022) (61) predicts that with the rapid growth of the global population, the demand for seafood will increase by 15% by 2053. However, the dual pressures of overfishing and environmental destruction have severely depleted marine resources, prompting global efforts to seek sustainable alternative protein sources to address this crisis.

This global issue also affects island nations, such as Taiwan, where the challenges faced by fisheries are particularly evident. Research indicates that, in recent years, the number of coastal fish species in Taiwan has significantly declined owing to overfishing, further exacerbating the pressure on local fishery resources (3, 4). Greenpeace (5) issued a warning that Taiwan’s fishery resources are experiencing irreversible deterioration owing to human activity. Under the dual pressures of internal and external environmental factors, exploring the feasibility of “plant-based seafood” as a novel food and its role in alleviating Taiwan’s resource crisis holds critical practical significance and research value. The promotion of plant-based seafood aligns with several United Nations Sustainable Development Goals (SDGs), including Goal 2 (Zero Hunger), Goal 12 (Responsible Consumption and Production), Goal 13 (Climate Action), and Goal 14 (Life Below Water).

Internationally, plant-based and cell-cultured seafood have gradually become an important trend in the development of sustainable diets. For example, the Netherlands has promoted cell culture technology and market development through a public-private collaboration model, aiming to reduce the pressure of traditional fishing on marine ecosystems (6). In the United States, venture capital has supported companies like “Good Catch,” which launched seafood alternatives based on plant proteins to meet consumers’ demand for healthy and sustainable foods (7). Plant-based seafood, marketed for its ability to replicate the distinctive flavors and textures of traditional seafood, has been proven to reduce environmental impact (8).

This industry is developing rapidly in Asia. For instance, Hong Kong’s Green Monday promotes plant-based products, such as fishmaws and fish balls, gradually bringing plant-based foods into the consumer’s spotlight. Taiwanese companies such as Grape King Bio, Chef’s Mart, and VEGUE have successively launched products such as vegan shrimp, plant-based fish fillets, and combination platforms for plant-based seafood, demonstrating Taiwan’s strong focus on innovative sustainable foods. However, systematic research in this emerging sector requires further investigation.

Although the plant-based food industry has rapidly developed internationally, the existing literature primarily focuses on technological innovations, production efficiency, or ingredient analysis related to plant-based seafood (9–11). To ensure the long-term success of sustainable food transitions, understanding consumers’ psychological mechanisms, value orientations, and behavioral decision-making processes is essential.

In Taiwan, where plant-based seafood is still in its early market stage, identifying the determinants that drive or hinder consumer acceptance is a critical research gap. However, research on consumer behavior, particularly regarding psychological mechanisms, values, and purchasing intentions, remains insufficient (12, 13). Furthermore, previous related studies have commonly adopted the Theory of Planned Behavior (TPB) as a framework for investigating consumer behavior (14).

However, TPB has limitations in measuring consumers’ moral motivations and the influence of personal norms. V**alue-Belief-Norm** (VBN) theory, which incorporates personal norms, can more effectively reflect an individual’s sense of responsibility toward the consequences of their own actions (15). Therefore, this study uses VBN theory as a foundation to explore how biospheric values, environmental beliefs, and personal moral norms influence Taiwanese consumers’ purchasing intentions for plant-based seafood products.

Furthermore, consumer psychological factors, such as Food Neophobia (FN) and Technological Trust (TT), have also been proven to play an important role in the market for novel foods (16–18). Research shows that resistance to novel foods is one of the reasons many consumers exhibit low acceptance of plant-based foods, which may stem from skepticism regarding the source of the food or distrust in its flavor and texture (7). When consumers have low trust in technological innovation, such distrust further increases psychological barriers to purchasing novel foods.

Therefore, this study simultaneously incorporated FN and TT as additional variables designed to analyze how contextual barriers and confidence mechanisms jointly influence consumer decisions alongside internalized moral motivations. We propose that FN and TT act as critical psychological factors that directly influence purchase and advocacy intention, offering a genuine theoretical contribution by extending the explanatory power of the traditional VBN model to account for affective barriers and cognitive trust in novel food contexts.

Based on this background, this study seeks to answer the following research questions:

1. Within the VBN theoretical framework, how do consumers’ biospheric values, environmental moral responsibility, and personal norms influence their purchase and advocacy intention for plant-based seafood products?
2. How does FN influence consumers’ purchase and advocacy intention toward plant-based seafood products?
3. Does TT influence consumers’ purchase and advocacy intention for plant-based seafood products?

This study contributes to both theory and practice. It employs VBN theory to investigate consumption behavior related to plant-based seafood, expanding the application of consumer psychology and environmental behavior theories. It also integrates FN and TT mechanisms to enhance the explanatory power of existing literature. Practically, the findings provide insights into strengthening Taiwan’s novel food market strategies, and offer empirical recommendations for producers, government policymakers, and consumer education. These contributions support the transition toward sustainable diets and marine resource conservation.

## 2 Literature review and hypothesis development

### 2.1 The value-belief-norm theory

The Value-Belief-Norm (VBN) theory, proposed by Stern (19), serves as the foundational psychological model for this study, aiming to explore the drivers of individuals’ environmentally significant behavior. The VBN framework models a sequential causal chain: Values→Beliefs→Norms→Behavior. This chain posits that deep-seated values influence broad environmental beliefs, which subsequently activate specific personal moral norms, ultimately leading to pro-environmental behaviors.

In recent years, this theory has been widely applied in various fields, such as low-carbon tourism (20), waste sorting (21), and organic food consumption (22), demonstrating its universality and effectiveness in explaining environmental behavior. We apply this core structure to investigate the psychological antecedents of purchasing plant-based seafood, a critical sustainable consumption choice.

#### 2.1.1 Biospheric values

In alignment with the VBN model, values are considered deep, fundamental drivers of behavior that determine individuals’ general attitudes and actions toward the environment (19). Furthermore, Feather (23) argued that values serve as an important foundation for behavior prediction and profoundly impact the formation of behavioral logic.

Biospheric values (BV), which reflect deep and abstract value orientations emphasizing concern for the well-being of nature and the sustainability of ecosystems (24), are hypothesized to be the initial inputs in the VBN causal chain. Chen and Chang (62) pointed out that when consumers perceive products as having a higher green perceived value, their attitudes toward environmentally friendly products improve.

According to Stern’s theoretical sequence, these values significantly influence the subsequent formation of environmental consciousness and beliefs. Therefore, we examined how consumers’ biospheric values lead to their environmental beliefs (H1).

*H1*: Biospheric values have a significant positive effect on consumers’ environmental beliefs for plant-based seafood.

#### 2.1.2 Environmental beliefs

Environmental beliefs (EB) refer to individuals’ cognitive evaluations of environmental issues, including general environmental worldviews and the ascription of personal responsibility to address them. EB explicitly represents the cognitive dimension within the VBN framework. Stern et al. (19) specified that environmental beliefs are composed of the New Environmental Paradigm (NEP), which reflects general attitudes toward issues such as climate change or resource depletion, and Ascription of Responsibility (AR), which denotes an individual’s sense of personal responsibility for engaging in environmental protection behaviors. These beliefs function as a critical cognitive link in the VBN chain, translating abstract values into an awareness of consequences and shared responsibilities. Tsitseli and Prodromitis (25) further highlight that beliefs often drive the formation of moral norms, especially in contexts with strong ideological influences or heightened issue awareness. Consequently, they drive the formation of the moral obligations that follow. We tested the influence of environmental beliefs on personal moral norms (H2).

*H2*: Environmental beliefs have a significant positive effect on consumers’ personal norms for plant-based seafood.

#### 2.1.3 Personal norms

Personal norms (PN) constitutes the final psychological step of the VBN sequence before the behavior. PN refer to an individual’s self-imposed sense of moral obligation and responsibility to take specific actions. This construct is essential because, compared to other constructs, personal norms exhibit significant predictive power for specific environmentally significant behaviors (26, 27). For example, viewing the purchase of plant-based or cell-cultured foods as a moral responsibility to reduce environmental impact often drives individual behavior (28).

Following the established VBN link (19), we examined how this internalized moral obligation motivates the specific action of purchasing plant-based seafood (H4).

#### 2.1.4 The mediating role of values and beliefs

The integrity of the VBN framework depends on an established mediating pathway. Stern et al. (29) emphasized that the core mechanism driving moral norms is the combination of values and beliefs, where values exert an indirect influence on moral norms through environmental beliefs. This mediating effect reveals the cognitive pathway through which values are transformed into behavioral responsibilities. Our model strictly adheres to this foundational principle by hypothesizing that environmental beliefs mediate the relationship between biospheric values and personal moral norms (H3), such that

*H3*: Environmental beliefs mediate the relationship between biospheric values and personal norms.

### 2.2 Purchase and advocacy intention

Purchase intention (PI) is a widely used indicator for assessing consumers’ willingness to engage in purchase-related behavior and for approximating potential market acceptance (30, 31). In the context of novel foods, particularly plant-based and cell-cultured seafood, purchase intention is frequently considered as a significant basis for measuring market potential (32). Furthermore, Bryant and Sanctorum (16) asserted that purchase intention is influenced by multiple psychological factors such as FN, TT, and environmental values, indicating the importance of an in-depth investigation into this topic. However, sustainable consumption intentions may encompass not only direct purchasing willingness but also broader supportive and advocacy-oriented behaviors. Accordingly, in the present study, the outcome construct is conceptualized as Purchase and Advocacy Intention (PAI), reflecting a broader intention to support plant-based seafood through both purchasing and promotional engagement. Therefore, this study proposes the following hypotheses. Therefore, this study hypothesizes the following.

*H4*: Personal norms have a significant positive effect on consumers’ purchase and advocacy intention for plant-based seafood.

### 2.3 Food neophobia and technological trust

While VBN theory effectively explains individuals’ internal moral motivations for pro-environmental behavior, consumers’ decisions regarding novel foods are also heavily influenced by context-specific psychological and situational factors. As noted by Laureati et al. (33), psychological and contextual determinants, such as FN, disgust, familiarity, and social context, are often more decisive in shaping consumer acceptance of novel foods. Consequently, effective strategies to promote novel food adoption must address both intrinsic moral values and psychological barriers.

FN and TT play significant roles in shaping consumers’ willingness to adopt innovative or unfamiliar food products (16–18). To extend the explanatory scope of the VBN framework, this study incorporates FN and TT as additional psychological constructs that complement the VBN-based explanation of consumers’ purchase intentions for plant-based seafood products.

Previous research has emphasized that consumer responses to sustainable or alternative foods are often driven not only by moral concerns, but also by perceptions of safety, familiarity, and confidence in production technologies (17). Therefore, FN and TT function not only as situational antecedents, but also as key psychological drivers that directly influence the final behavioral outcome within the extended VBN structure.

In the context of novel and alternative foods, FN and TT represent two distinct psychological factors that complement the VBN framework by accounting for consumers’ risk-related and technology-related evaluations beyond moral considerations. Specifically, FN captures affective resistance toward unfamiliar foods, which directly reduces purchase intention, whereas TT reflects consumers’ confidence in food production technologies, which directly enhances purchase intention by alleviating perceived uncertainty and risk. FN in this study refers to trait food neophobia, defined as an individual’s relatively stable, affective tendency to avoid or feel apprehensive toward novel foods due to uncertainty or perceived risk (34, 35). This construct reflects a relatively stable psychological trait that often influences consumers’ acceptance of innovative foods such as plant-based or lab-cultured products.

FN significantly influences consumer attitudes toward novel foods, particularly in contexts with higher perceived risks (36). Siegrist and Hartmann (18) further indicated that when brand trust or food information transparency is lacking, consumers with high FN show significantly reduced purchase intentions for plant- and cell-cultured foods. Within the context of novel and alternative foods, FN represents an affective barrier that directly reduces consumers’ willingness to engage with unfamiliar products, thereby negatively influencing purchase and advocacy intention. Therefore, this study hypothesizes the following.

*H5*: FN has a significant negative effect on consumers’ purchase and advocacy intention for plant-based seafood.

TT refers to an individual’s confidence in a technology or technological system (37). At its core, this construct reflects an individual’s evaluation of whether a technology is dependable and operates reliably while also encompassing a normative belief that users are entitled to expect an appropriate level of performance and reliability from the technology (38).

In the context of food technology, TT represents consumer confidence in the safety, transparency, and reliability of production processes, particularly those involving innovative or biotechnological foods (17). Building on this perspective, this study conceptualizes TT as a key psychological mechanism that influences consumers’ purchase intentions for plant-based seafood.

Siegrist and Hartmann (18) indicate that when consumers have low trust in biotechnology or laboratory cultivation techniques, they develop health concerns about food, which subsequently inhibits their willingness to accept it. Furthermore, Fu (39) suggested that transparent technological information can effectively enhance consumer confidence, thereby promoting the intention to purchase products in the market.

Within the context of novel and alternative foods, TT functions as a cognitive enabler that directly enhances consumers’ purchase intentions by reducing perceived uncertainty and risk associated with innovation-driven food technologies. Rather than shaping moral obligations, TT influences consumers’ evaluations of product safety, reliability, and transparency, thereby increasing their willingness to accept and purchase plant-based seafood products. Accordingly, this study proposes the following hypothesis.

*H6*: TT has a significant positive effect on consumers’ purchase and advocacy intention for plant-based seafood.

Furthermore, given their contrasting psychological roles as an affective barrier (FN) and a cognitive enabler (TT) in the context of novelty and perceived risk, this study examines their respective contributions to consumers’ purchase and advocacy intention for plant-based seafood. By incorporating both constructs into an extended VBN framework, the analysis clarifies how FN and TT provide additional explanatory power beyond value–belief–norm factors in understanding consumers’ responses to novel food products.

### 2.4 Research framework

**Figure 1:**
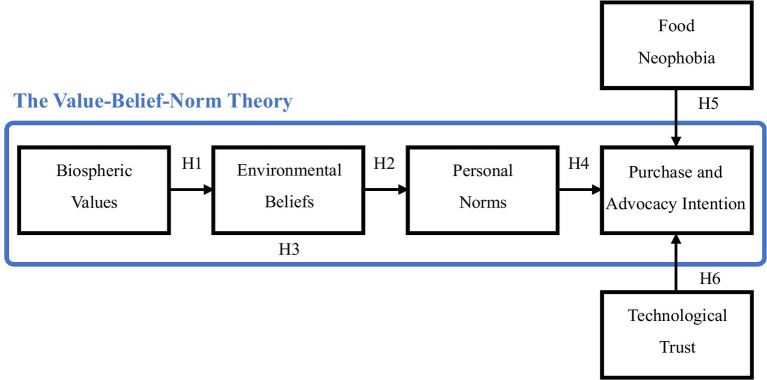
Conceptual framework and hypotheses of the study. Source(s): Authors’ own work.

Drawing from the VBN, this study developed an integrated theoretical framework to examine Taiwanese consumers’ purchase and advocacy intention for plant-based seafood. The framework extends the traditional VBN model by incorporating two critical psychological constructs - FN and TT - as additional predictors to enhance the model’s explanatory power in the context of novel food acceptance. This comprehensive framework advances our understanding of the complex relationships among consumers’ environmental values, beliefs, and behavioral intentions. The conceptual model is illustrated in Figure 1.

## 3 Materials and methods

### 3.1 Questionnaire design

The questionnaire consisted of two sections. The first part measured the research constructs, adhering strictly to the VBN sequential structure proposed by Stern (19), while utilizing measurement items adapted from recent, contextually validated literature. This approach was necessary because the original VBN scales were developed to address broad environmental concerns, whereas our study specifically focused on novel food acceptance (plant-based seafood).

To ensure the relevance and predictive validity of the constructs examined in this study, all measurement scales were drawn from recent sustainable consumption research, in which they were empirically validated and conceptually refined. Biospheric values were assessed using four items adapted from Chunhua et al. (40), and environmental beliefs were measured using six items derived from Trautwein et al. (41) and Farm et al. (42). Personal moral norms were evaluated using three items adapted from Yeow and Loo (43). FN was measured using four items selected from the Food Neophobia Scale (FNS) developed by Pliner and Hobden (35), following the wording and application in Dursun and Gümüş (44). TT was assessed using three items adapted from Ali et al. (45). Finally, Purchase and Advocacy Intention (PAI) was measured using five items adapted from Chang et al. (46) and Nguyen et al. (47). The indicators capture both transactional purchase commitment (e.g., willingness to prioritize purchasing and increase purchase frequency) and supportive or advocacy-oriented intentions (e.g., recommending or promoting plant-based seafood to others). Accordingly, the construct is conceptualized as reflecting a broader intention to support plant-based seafood, encompassing both personal purchasing willingness and promotional advocacy behaviors. Collectively, these validated scales ensured robust measurement quality and enhanced the rigor and comparability of the study’s empirical findings.

All items were originally developed in English and translated into Chinese, following a standard back-translation procedure to ensure semantic equivalence. Minor wording adjustments were made to fit the specific context of this study. All items were measured on a seven-point Likert scale ranging from 1 (“strongly disagree”) to 7 (“strongly agree”), with higher scores indicating stronger agreement. The second part of the questionnaire collected demographic information including sex, age, education level, monthly income, occupation, and dietary habits.

The empirical success of this scale adaptation was confirmed by the subsequent measurement model assessment; all constructs demonstrated strong internal consistency, with Cronbach’s coefficients surpassing 0.7, and average variance extracted (AVE) values exceeding 0.5 (see Section 4.1). This methodological approach ensures that the measurement tool maintains conceptual fidelity to Stern’s VBN structure, while achieving the robust contemporary psychometric performance necessary for modeling novel food behavior.

### 3.2 Sample and data collection

Questionnaires were distributed through the Internet and social media channels to collect the research data. Prior to distributing the formal questionnaire, a pilot test was conducted to enhance the reliability of the questionnaire scale through respondents’ feedback. Fifty valid responses were collected. Reliability measures were used to assess the stability and consistency of construct measurements. The formal questionnaire was distributed from January 1, 2025, to February 23, 2025. The researchers distributed the questionnaires via word of mouth, Facebook, Instagram, and Line groups and communities.

The study was conducted in accordance with the Declaration of Helsinki. Ethical review and approval were waived for this study by the Institutional Review Board of Chung Shan Medical University Hospital (under regulation SOP034) for anonymous, non-invasive consumer surveys. Informed consent was obtained digitally from all subjects involved in the study. The front page of the questionnaire clearly explained the research purpose, confirmed that participation was voluntary and anonymous, and stated that proceeding with the survey indicated consent. Data were stored on a secure, password-protected server and accessible only to the research team to ensure privacy.

In total, 345 questionnaires were collected. Data screening procedures were conducted prior to analysis to ensure response quality.

As the online survey system required respondents to complete all items before submission, incomplete questionnaires and missing data were not possible.

Response quality was assessed using multiple criteria. First, 65 cases exhibiting clear straight-lining patterns (i.e., identical responses across multiple scale items) were excluded, as such patterns may indicate insufficient engagement with the survey. Second, duplicate submissions were minimized by restricting access to respondents logged into a Google account, with each account permitted to submit only one response. Email addresses were not collected or linked to responses, thereby preserving anonymity. No duplicate submissions were detected based on account-level and timestamp screening.

No formal attention-check items were included, and no predefined minimum completion-time threshold was imposed, given potential variability in individual reading speeds. We acknowledge the absence of these specific quality checks as a methodological limitation.

After applying these data-quality procedures, 280 valid responses were retained for statistical analysis. The demographic characteristics of the final sample are presented in Table 2.

### 3.3 Methods of data analysis

Quantitative research methods were utilized in this study, with data collected through questionnaire surveys and analyzed using IBM SPSS Statistics 27 and AMOS 28. The statistical techniques applied included descriptive statistics, such as frequency distributions, percentages, means, and standard deviations, as well as reliability and validity assessments. Additionally, structural equation modeling employing Maximum Likelihood estimation was conducted to evaluate the causal relationships and overall fit of the proposed model, thereby confirming the research hypotheses outlined in the study.

## 4 Analysis and results

### 4.1 Reliability and validity assessment of the measurement model

Robustness of the measurement scale was evaluated using comprehensive reliability and validity analyses. As presented in Table 1, reliability testing revealed Cronbach’s *α* coefficients surpassing 0.7 across all dimensions, satisfying Bernstein and Nunnally’s (48) recommended threshold and confirming the scale’s internal consistency. Convergent validity assessment followed Fornell and Larcker’s (49) framework, yielding three key findings.

**Table 1:** Results of the factor loading, reliability, and validity.

Variables	Items	Standardized Factor Loadings	CR	AVE	Cronbach’s α
Biospheric values (BV)	1. I believe that pollution reduction is a fundamental human responsibility.	0.853***	0.896	0.682	0.837
	2. I believe that respecting the Earth is a basic principle of human behavior.	0.822***			
	3. I believe that humans should strive to live in harmony with nature.	0.789***			
	4. I believe protecting the environment is a collective responsibility.	0.839***			
Environmental beliefs (EB)	1. I believe that human activity can cause severe environmental damage.	0.691***	0.910	0.631	0.829
	2. I believe that my current efforts in environmental protection are still insufficient.	0.765***			
	3. I believe we will face major ecological disasters if no action is taken.	0.708***			
	4. I believe that I have an obligation to conserve and be responsible for energy use.	0.858***			
	5. I believe that I have an inescapable responsibility for energy depletion.	0.851***			
	6. I believe that I will contribute to mitigating global warming.	0.872***			
Personal norms (PN)	1. I believe that I have a moral obligation to purchase plant-based seafood.	0.924***	0.946	0.853	0.907
	2. I am willing to make greater efforts to purchase plant-based seafood.	0.949***			
	3. If I choose not to purchase plant-based seafood, I feel guilty and self-reproachful.	0.897***			
Food neophobia (FN)	1. I am skeptical of the safety of novel foods.	0.701***	0.860	0.610	0.749
	2. I will not try food if I am unclear about its ingredients.	0.651***			
	3. I experience a notable fear of food I have never encountered.	0.875***			
	4. I am selective about food choices and do not readily accept novel foods.	0.871***			
Technological trust (TT)	1. I believe that the production technology for plant-based seafood complies with legal regulations.	0.913***	0.955	0.875	0.926
	2. I believe that the production technology of plant-based seafood adequately considers the technical standards and ensures consumer safety.	0.961***			
	3. I believe that the production technology for plant-based seafood is safe and reliable for my health.	0.931***			
Purchase and advocacy intention (PAI)	1. I support plant-based seafood as a novel food source.	0.765***	0.949	0.787	0.932
	2. I am willing to prioritize purchasing plant-based seafood.	0.895***			
	3. I am willing to introduce the advantages and characteristics of plant-based seafood to my family and friends.	0.940***			
	4. I will recommend that others purchase plant-based seafood.	0.919***			
	5. In the future, I will increase the frequency of purchasing plant-based seafood.	0.907***			

***p < 0.001. Source(s): Authors’ own work.

**Table 2:** Demographic characteristics of the respondents.

*N* = 280	Item	Population	Percentage (%)
Have you ever purchased plant-based food?	Yes	155	55.4
	No	125	44.6
Have you ever purchased plant-based seafood?	Yes	65	23.2
	No	215	76.8
Gender	Male	137	48.9
	Female	143	51.1
Age	21–30 years	76	27.1
	31–40 years	71	25.4
	41–50 years	69	24.6
	51–60 years	49	17.5
	60 years and above	15	5.4
Level of Education	Middle school or below	5	1.8
	High school/vocational	98	35.0
	College/university	132	47.1
	Master’s or above	45	16.1
Monthly personal income	Less than NTD 20,000 (inclusive)	55	19.6
	NTD 20,001 ~ 40,000	140	50.0
	NTD 40,001 ~ $60,000	45	16.1
	NTD 60,001 ~ 80,000	15	5.4
	NTD 80,001 ~ 100,000	20	7.1
	Above NTD 100,001	5	1.8
Occupation	Student	70	25.0
	Army, civil service, and education	20	7.1
	Service industry	90	32.1
	Freelance	10	3.6
	Traditional manufacturing	35	12.5
	Other	55	19.6
Dietary habits	Omnivorous	191	68.2
	Flexitarian	89	31.8

Source(s): Authors’ own work.

First, all items demonstrated standardized factor loadings greater than 0.5, confirming their effectiveness in representing their respective constructs. Second, the AVE values exceeded the 0.5 threshold, validating appropriate construct variance sharing. Third, composite reliability (CR) measurements surpassed 0.6, fulfilling the internal consistency requirements established by Bagozzi and Yi (50). Collectively, these results substantiate the reliability and convergent validity of the measurement scale, and establish a sound foundation for subsequent analytical procedures. Additionally, robustness checks using HTMT and CLF analyses confirmed adequate discriminant validity and minimal impact of common method variance (see Appendix A).

Discriminant validity assessment of the measurement model was conducted, and the results are presented in Table 3. The evaluation followed Fornell and Larcker’s (49) methodology, which involves comparing each construct’s AVE square root with the inter-construct correlation coefficients. The criterion for establishing discriminant validity requires a construct’s AVE square root to exceed its correlation values with the other constructs, thereby confirming its distinct nature.

**Table 3:** Correlation coefficients alongside the square root of AVE.

Variables	Mean	Standard Deviation	1	2	3	4	5	6
1. Biospheric Values	6.1786	0.8851	**0.826**					
2. Environmental Beliefs	5.8542	0.8779	0.724**	**0.794**				
3. Personal Norms	3.9583	1.6164	0.368**	0.466**	**0.924**			
4. Food Neophobia	4.7277	1.2088	−0.350	−0.380	0.091	**0.781**		
5. Technological Trust	5.0298	1.3480	0.360**	0.444**	0.327**	−0.014	**0.936**	
6. Purchase and Advocacy Intention	4.2000	1.3881	0.319**	0.218**	0.534**	0.001	0.646**	**0.887**

The bold values on the diagonal represent the square root of AVE for each variable. ** p < 0.05. Source(s): Authors’ own work.

Table 3 reveals that all constructs exhibited AVE square roots superior to their corresponding interconstruct correlation coefficients, thus establishing robust discriminant validity. This validation validates the measurement instrument’s capacity to effectively distinguish between different latent variables, thereby strengthening the overall methodological rigor and credibility of the research outcomes.

### 4.2 Model fit assessment

Maximum Likelihood (ML) estimation was employed to specify and evaluate the structural model and test the hypothesized relationships within the proposed theoretical framework. Model fit evaluation was conducted using a combination of absolute, incremental, and parsimony-adjusted indices, consistent with the established recommendations in the structural equation modeling literature (51).

The results indicated that all fit indices satisfied or exceeded the widely accepted cut-off criteria, thereby supporting an overall satisfactory fit of the model to the data. Specifically, the values obtained were as follows: chi-square to degrees of freedom ratio (χ^2^/df) = 4.977 (<5), goodness-of-fit index (GFI) = 0.922 (>0.90), root mean square residual (RMR) = 0.079 (<0.08), standardized root mean square residual (SRMR) = 0.078 (<0.08), root mean square error of approximation (RMSEA) = 0.079, indicating acceptable fit (90% CI [0.072, 0.086]), normed fit index (NFI) = 0.953 (>0.90), comparative fit index (CFI) = 0.975 (>0.90), incremental fit index (IFI) = 0.977 (>0.90), and parsimony-adjusted normed fit index (PNFI) = 0.766 (>0.50).

Taken together, these indices demonstrate that the structural model possesses strong goodness-of-fit across multiple dimensions—absolute, incremental, and parsimony-adjusted—providing a robust empirical basis for subsequent hypothesis testing and exploration of the structural relationships among the latent variables.

### 4.3 Path analysis and model testing results

The path analysis and hypothesis-testing outcomes are presented in Table 4. The findings revealed that biospheric values significantly and positively affect consumers’ environmental beliefs (*β* = 0.914, *p* < 0.001). Environmental beliefs had a significantly positive impact on personal moral norms (*β* = 0.679, *p* < 0.001). Personal moral norms had a significant positive effect on consumers’ intentions to purchase plant-based seafood (*β* = 0.596, *p* < 0.001). FN showed no significant relationship with intention to purchase plant-based seafood (*β* = −0.017, *p* = 0.754). TT emerged as a significant positive predictor of intention to purchase plant-based seafood (*β* = 0.349, *p* < 0.001). Based on these results, H1, H2, H4, and H6 were empirically supported, but H5 was not substantiated.

**Table 4:** Findings from the path analysis and validations of hypotheses.

Hypothesized paths	Unstandardized coefficient	S.E.	C.R.	*p*	Standardized coefficients	Verification results
H1: BV → EB	0.331	0.048	6.924	***	0.914	Supported
H2: EB → PN	2.387	0.782	3.052	***	0.679	Supported
H4: PN → PAI	0.675	0.064	10.476	***	0.596	Supported
H5: FN → PAI	−0.029	0.093	−0.313	0.754	−0.017	Unsupported
H6: TT → PAI	0.487	0.076	6.449	***	0.349	Supported

*** *p* < 0.001. Source(s): Authors’ own work.

As a brief robustness check, the structural relationships were examined separately for respondents with and without prior experience purchasing plant-based foods (*n* = 155 vs. *n* = 125). Across both subgroups, biospheric values positively predicted environmental beliefs (*β* = 0.809 vs. 0.404, *p* < 0.001), and environmental beliefs positively predicted personal norms (*β* = 0.658 vs. 0.381, *p* < 0.001).

Personal norms and technological trust remained significant positive predictors of purchase and advocacy intention in both groups. Technological trust showed a stronger association among experienced consumers (*β* = 0.665 vs. 0.281, *p* < 0.001), whereas personal norms appeared relatively more influential among those without prior experience (*β* = 0.266 vs. 0.418, *p* < 0.001).

Food neophobia was non-significant among respondents with prior plant-based food experience (*β* = 0.083, *p* = 0.079), but negatively associated with purchase and advocacy intention among those without prior experience (*β* = −0.365, *p* < 0.001). This pattern suggests that neophobia may operate primarily as a barrier during the initial adoption stage, while its influence diminishes once consumers gain familiarity with plant-based products. In other words, prior experiential exposure may attenuate the psychological resistance typically associated with novel foods. This finding helps explain the non-significant direct effect of food neophobia observed in the full-sample SEM and indicates that neophobia may function as a conditional constraint rather than a universally stable predictor of purchase and advocacy intention.

Overall, the direction of effects was consistent with the main SEM findings, supporting the robustness of the core value–belief–norm and trust-based mechanisms across subgroups.

### 4.4 Analysis of mediating relationships between biospheric values and environmental beliefs

To examine the mediating relationships between biospheric values (BV), environmental beliefs (EB), and personal norms (PN), a bias-corrected bootstrapping approach was employed as the primary analytical method, given its robustness and superior statistical power in detecting indirect effects. Traditional regression-based mediation tests, such as the procedure proposed by Baron and Kenny (52), may suffer from limitations, including low statistical power and limited sensitivity in identifying indirect effects. Accordingly, the mediation effects in the present study were evaluated using a bootstrapping approach.

Using AMOS 28, a bias-corrected bootstrapping analysis with 5,000 resamples and a 95% confidence interval was conducted, and the results are shown in Table 5. The analysis indicated that the standardized indirect effect of BV on PN via EB was 0.621, and the 95% bias-corrected confidence interval (BC CI = [0.197, 1.362]) did not include zero, indicating a statistically significant mediating effect of EB. Meanwhile, the direct effect of biospheric values (BV) on personal norms (PN) was not statistically significant (estimate = −0.156, *p* = 0.546). This result supports a full mediation pattern, indicating that the influence of biospheric values on personal norms is fully transmitted through environmental beliefs (EB), with no residual direct effect remaining.

**Table 5:** Bias-corrected bootstrapping results for the mediating effect.

Path	Estimate	95% Confidence interval		
		BC/PC *p*-value	BC	PC
Indirect effect				
BV → EB → PN	0.621	0.005/0.006	0.197 ~ 1.362	0.184 ~ 1.306
Direct effect				
BV → EB	0.914	< 0.001/< 0.001	0.833 ~ 0.968	0.827 ~ 0.965
BV → PN	−0.156	0.546/0.521	−0.829 ~ 0.344	−0.854 ~ 0.334
EB → PN	0.679	0.006/0.006	0.205 ~ 1.364	0.205 ~ 1.366
Total effect				
BV → PN	0.465	< 0.001/< 0.001	0.345 ~ 0.570	0.339 ~ 0.566

The values in table are standardized regression coefficients (β). Source(s): Authors’ own work.

Overall, the bootstrapping results provide robust empirical support for the mediating role of EB between BV and PN, thereby supporting H3.

## 5 Discussion

### 5.1 Theoretical implications

This study investigated consumers’ purchase and advocacy intentions toward plant-based seafood within the VBN theoretical framework while incorporating FN and TT as contextual psychological variables. The results reaffirm the robustness of the VBN framework in explaining sustainable consumption behavior and suggest theoretical extensions that account for contextual trust and emotional responses.

First, biospheric values significantly and positively influenced environmental beliefs, which, in turn, strengthened personal moral norms and purchase and advocacy intention. These findings align with VBN theory [(15, 53–56); Čapienė et al., 2023], reinforcing the idea that moral decision-making in sustainability contexts arises from an internalized sense of environmental responsibility.

Moreover, the mediating role of environmental beliefs in the relationship between biospheric values and personal moral norms highlights the cognitive pathway through which value orientation is translated into moral obligation. Therefore, strengthening environmental beliefs is a crucial factor in promoting consumers’ intentions to engage in sustainable consumption behavior (57). This finding underscores the importance of environmental cognition as a bridge between abstract ecological values and specific behavioral intentions, offering a potential refinement to VBN theory by emphasizing belief salience as a dynamic process rather than a static construct.

Second, Food Neophobia (FN) did not significantly influence purchase and advocacy intention (H5 was not supported) in the full-sample SEM, which partially diverges from prior findings (58). This result should be interpreted with caution, as it does not imply that plant-based seafood is universally perceived as non-novel. Indeed, plant-based seafood remains an emerging category in the Taiwanese market, and 76.8% of respondents reported never having purchased such products.

To further examine this issue, we conducted a robustness analysis by separating respondents based on prior experience purchasing plant-based foods. The results revealed a meaningful heterogeneity pattern: FN was non-significant among consumers with prior plant-based food experience, but showed a significant negative association with purchase intention among those without prior experience.

This pattern suggests that neophobia may function primarily as a barrier at the early stage of adoption, exerting stronger influence among consumers who lack prior experiential exposure. Once individuals gain familiarity with plant-based foods, the psychological salience of novelty-based apprehension appears to diminish, reducing the impact of FN on intention formation. In this sense, experiential familiarity may attenuate affective resistance, while cognitive factors such as technological trust remain influential across groups.

At the same time, the absence of direct measures of perceived unfamiliarity or product-specific novelty limits our ability to test this mechanism more directly. Future research should incorporate explicit measures of perceived novelty and stage-of-adoption dynamics to better understand how neophobia operates across different consumer segments. Such refinement would further clarify the conditional role of affective resistance within value-based behavioral models.

This conditional effect is consistent with recent findings suggesting that psychological antecedents of sustainable food adoption may operate differently depending on consumers’ prior experience and familiarity with alternative food products. For example, Tunca et al. (59) show that affective and cognitive components in behavioral intention models such as the TPB can exhibit varying degrees of influence depending on food-related apprehension and contextual familiarity, implying that neophobic responses tend to be more salient among less experienced consumers.

Third, TT has a strong positive influence on purchase and advocacy intention, which aligns with Bryant and Sanctorum’s (16) findings, and suggests that trust in technological innovation functions as a contextual enabler in the VBN framework. While moral norms explain the motivation to act sustainably, TT appears to translate moral intentions into concrete purchasing behavior by reducing perceived uncertainty and increasing confidence in product safety and transparency. This finding provides an important theoretical contribution, indicating that situational trust mechanisms may serve as complementary pathways bridging moral motivation and purchase and advocacy intention, an association that is not traditionally emphasized within the VBN framework.

In summary, this study extends the theoretical reach of the VBN framework by illustrating how contextual confidence (TT) and affective resistance (FN) interact with the moral and cognitive dimensions of sustainable behavior. These insights suggest that future research should further examine the interplay between moral values, trust mechanisms, and affective barriers to explain behavioral inconsistencies in sustainable food consumption. It should be noted that the purchase and advocacy intention construct in this study reflects consumers’ stated willingness and support for plant-based seafood rather than actual purchasing behavior, and an intention–behavior gap may therefore exist.

### 5.2 Management implications

These findings indicate that consumers’ purchasing decisions are influenced by their values, environmental beliefs, personal moral norms, and TT. These implications are grounded in psychological determinants of intention formation identified within the VBN framework. Accordingly, they should be interpreted as strategies to activate value-based motivations and strengthen perceived credibility, rather than as direct prescriptions for product optimization, pricing strategies, or market-level forecasting. Therefore, food industry operators and relevant policymakers can adjust their management and marketing strategies in the following ways:

1) Strengthening environmental education and promotion: Given that biospheric values significantly influence environmental beliefs and personal moral norms, it is essential to reinforce consumers’ awareness of environmental issues through education and public engagement. Government agencies and nonprofit organizations can implement campaigns that emphasize the ecological and moral benefits of sustainable seafood alternatives. Such efforts can enhance consumers’ sense of environmental responsibility and strengthen the value–belief–norm chain that motivates sustainable consumption.
2) Enhanced TT and information transparency: As TT has a strong positive influence on consumers’ purchase and advocacy intention, marketing strategies should focus on reinforcing transparency and technological credibility. The food industry can increase consumer confidence by disclosing information about ingredient sourcing, production technologies, and safety standards. Third-party certifications and traceable labeling systems may serve as mechanisms to enhance perceived technological credibility.
3) Eliminating consumer concerns about novel foods: Although this study found that FN does not significantly affect purchase and advocacy intention, it is still recommended that businesses promote plant-based seafood through food tasting, consumer experiences, and word-of-mouth marketing to reduce psychological barriers among potential consumers and improve product acceptance. Such experiential strategies may work through both reduced neophobia and improved sensory evaluations; however, the present study only captures the former psychological pathway and does not empirically model sensory attributes.
4) Strengthening moral norms and sustainable consumption behavior: Since personal moral norms are found to significantly influence purchase and advocacy intention, companies can design marketing communications that evoke ethical and moral considerations. Highlighting animal welfare, ocean conservation, and environmental protection within brand storytelling can activate consumers’ moral self-concept and promote long-term sustainable consumption orientations and purchase-related intentions.
5) Promoting government-business cooperation: The government can encourage enterprises to develop more plant-based seafood products through policies such as research and development subsidies, tax reductions, or certification incentives to create supportive institutional environments that may facilitate intention translation. Businesses can collaborate with the government to promote food standards and certification systems, ensure product quality and build consumer trust.

This study provides management and policy insights into the development of plant-based seafood products. In the future, businesses can enhance market acceptance and promote sustainable consumption behavior by strengthening environmental education, information transparency, and consumer trust.

## 6 Conclusion

Based on the model verification results, this study presents the following discussion, conclusions, and recommendations:

### 6.1 Research conclusions

The research findings indicate that consumers’ biospheric values, environmental beliefs, personal moral norms, and TT are key factors that influence their purchasing decisions for plant-based seafood products. As plant-based seafood remains an emerging food category with market acceptance that is still in development, consumer understanding of its product quality, safety, and environmental benefits remains incomplete. Therefore, enhancing public awareness and trust in these products is crucial for expanding the plant-based seafood market.

Currently, plant-based seafood has a limited recognition in the Taiwanese market. While some consumers are aware of its environmental advantages, trust in its production processes and food technology still needs improvement, and these products remain relatively limited compared with plant-based meat and dairy alternatives. Furthermore, many people have an incomplete knowledge of plant-based seafood. Some respondents reported consuming vegetarian seafood, a subset of plant-based seafood, indicating that many consumers were unaware that they had previously consumed or purchased these products.

It is recommended that businesses enhance information transparency through third-party certifications, disclosure of product ingredients and manufacturing processes, and environmental benefit labeling, to reduce consumer uncertainty and further increase purchase and advocacy intention. In addition, the government can provide policy guidance to help establish standardized food certification and labeling systems to improve market regulations and consumer confidence.

Moreover, this study found that personal moral norms played a significant role in plant-based seafood consumption decisions, indicating that consumers’ environmental awareness and moral values influence their acceptance of plant-based seafood. Therefore, when promoting plant-based seafood, businesses can emphasize their positive contributions to environmental and marine protection to attract groups that value sustainable development and ethical consumption. Through the coordination of educational promotion, marketing strategies, and government policies, the development of the plant-based seafood market can be accelerated, further promoting green consumption behavior.

### 6.2 Research limitations and future research directions

Despite providing insight, this study has several areas for future research. First, future studies could explore consumers’ behavioral intentions and preferences for plant-based seafood across different cultural contexts. Second, while this study used the VBN theory to analyze consumer behavioral intentions toward plant-based seafood, future research could incorporate more behavioral science theories, such as the TPB or Self-Determination Theory (SDT), to further explore the multiple factors influencing consumer behavior. Furthermore, future research could use more diverse research methods, such as eye tracking technology, to gain deeper insights into consumer decision-making processes and behavioral patterns in different contexts.

This study focused on psychological and value-based determinants of intention formation within a VBN-based framework. Product-level attributes such as sensory expectations (e.g., taste and texture), perceived naturalness or degree of processing, price, and product availability were beyond the scope of the present psychological-model design. These attributes are well-established determinants of alternative protein acceptance, and their exclusion implies that the current model explains psychological readiness and motivational drivers rather than downstream market outcomes or product-level trade-offs.

Accordingly, we explicitly frame this as a key scope boundary and limitation of the study. Theoretical and practical implications should therefore be interpreted at the level of internal motivations, beliefs, and value-based mechanisms, rather than as direct guidance for optimizing sensory characteristics, pricing strategies, or distribution systems of plant-based seafood products.

Future research would benefit from integrating value-based psychological mechanisms with systematic assessments of product attributes—such as sensory quality, perceived naturalness and processing, price sensitivity, and availability—to provide a more behaviorally and market-complete account of alternative protein adoption within sustainable diet transitions.

In addition, this study employed an online survey distributed through social media platforms, which constitutes a form of convenience sampling and may have involved self-selection bias. The sample was relatively younger and more highly educated than the general seafood-consuming population. While this sampling approach is common in research on emerging food products, it may limit the external validity and generalizability of these findings. Therefore, the results should be interpreted as reflecting the psychological mechanisms underlying purchase-related intentions within the sampled population, rather than the broader consumer population. Future research should consider employing probability-based sampling methods or mixed recruitment strategies to enhance representativeness.

In addition, this study did not explicitly examine demographic heterogeneity or measurement invariance across the population subgroups. The primary analytical focus was on the overall psychological mechanisms proposed by the VBN framework rather than on comparing structural relationships across demographic groups such as sex, age, income, or dietary type. Accordingly, demographic variables were not incorporated as control variables in the structural model, and formal measurement invariance tests were not performed. While demographic characteristics may influence consumers’ food-related decisions, incorporating multiple demographic controls or multi-group analyses could obscure the interpretation of the core theoretical pathways emphasized in this study. Future research should explicitly investigate demographic heterogeneity by employing multi-group structural equation modeling or moderation analyses, as well as test measurement invariance across key demographic segments to further assess the generalizability of the proposed model.

A key limitation concerns the operationalization of purchase and advocacy intention. The five-item scale used (adapted from (46, 47)) includes items that measure both direct purchasing commitment (e.g., “willing to prioritize purchasing plant-based seafood” and “increase the frequency of purchasing”) and broader behavioral support, such as advocacy (e.g., “willing to introduce the advantages” and “recommend that others purchase”) and general attitude (“I support plant-based seafood”). While the composite measure achieved strong psychometric 654 reliability (CR = 0.949, AVE = 0.787), we note that a sensitivity analysis restricting the PI construct to only the two core transactional items yielded substantially poorer overall model fit (CFI = 0.676; RMSEA = 0.176). This suggests the broader scale offers superior model stability for this context. Nevertheless, future research should consider restricting the PI construct to only core transactional intention items. This approach would ensure greater conceptual purity for modeling specific purchase behavior or, alternatively, model 658 advocacy/recommendation as a distinct post-intention outcome variable.

## Data Availability

The original contributions presented in the study are included in the article/Supplementary material, further inquiries can be directed to the corresponding author/s.
