# Supplementary material for: Seafood not from the sea: examining consumer behavioral intentions toward plant-based seafood
Source: Front Nutr. 2026 Mar 10;13:1782036. doi: 10.3389/fnut.2026.1782036 (PMC13008730; doi:10.3389/fnut.2026.1782036)
Supplement: Supplementary file 1 [file Table_1.docx]

**Appendix A** **: Robustness Checks**

**Table A1**. CLF Test Results

| Model | $x^{2}$(CMIN) | *df* | Δ$x^{2}$ (Δdf) | p-value |
| --- | --- | --- | --- | --- |
| Model 1 (CLF free) | 955.275 | 235 |  | <0.001 |
| Model 2 (CLF loadings=0) | 1294.02 | 260 | 338.745(25) | <0.001 |

Note: Δ*df* = 25 (number of observed indicators); Δ$x^{2}$ test significant indicates CLF free model fits significantly better than constrained model.

**Source(s):** Authors’ own work

To diagnose potential common method variance (CMV), we estimated a common latent factor (CLF) model in AMOS. The CLF was specified as a single latent factor loading on all 25 observed indicators used in the main SEM, in addition to their theorized latent constructs. For identification, the variance of the CLF was fixed to 1.0, and all CLF loadings were freely estimated in Model 1 (CLF included). In the constrained model (Model 2), the same CLF was retained but all of its loadings were fixed to zero, thereby removing its capacity to capture shared variance. The two models are summarized in Table A1.

Model 1 provided a significantly better fit than Model 2, Δ$x^{2}$(25) = 338.745, *p* < 0.001, indicating that the CLF accounts for a statistically detectable portion of shared variance among the indicators. Inspection of the standardized CLF loadings in Model 1 revealed a heterogeneous pattern: several items exhibited very small loadings (|λ| < 0.10), a number showed small-to-moderate loadings (0.10 ≤ |λ| ≤ 0.30), whereas five of the twenty-five items loaded more strongly on the CLF (|λ| > 0.50). This pattern suggests that shared method variance may affect certain indicators more than others.

To assess whether CMV materially biased the substantive relationships, structural path coefficients were compared across the two models. The key paths from PN and TT to PI changed only marginally when the CLF was included (|Δβ| ≤ 0.05), and the FN → PI path remained non-significant in both models. The BV → EB path showed a reduction in magnitude (from β = 0.914 to β = 0.680; |Δβ| = 0.234) but remained highly significant in both specifications.

Overall, these findings indicate that although some shared method variance is statistically detectable at the item level, the primary structural relationships remain largely stable across specifications, suggesting that CMV does not materially distort the substantive pattern of results observed in this study.

**Table A2**. Heterotrait–Monotrait (HTMT) ratio matrix

|  | BV | EB | FN | PI | PN | TT |
| --- | --- | --- | --- | --- | --- | --- |
| BV |  |  |  |  |  |  |
| EB | 0.845 |  |  |  |  |  |
| FN | 0.215 | 0.211 |  |  |  |  |
| PI | 0.363 | 0.284 | 0.098 |  |  |  |
| PN | 0.406 | 0.521 | 0.203 | 0.571 |  |  |
| TT | 0.391 | 0.498 | 0.118 | 0.698 | 0.349 |  |

**Source(s):** Authors’ own work

To complement the Fornell–Larcker criterion, discriminant validity was further assessed using the heterotrait–monotrait ratio of correlations (HTMT; Henseler et al., 2015), computed in SmartPLS 4. Following Henseler et al. (2015), a conservative threshold of 0.85 was adopted, while values below 0.90 are generally considered acceptable.

As shown in Table A2, all HTMT values were below 0.85 except for the BV–EB pair (0.845), which remains below the more liberal 0.90 cutoff. Importantly, the HTMT value between Food Neophobia (FN) and Technological Trust (TT) was 0.118, well below established thresholds, indicating strong discriminant validity between these constructs.

These results provide empirical support that the constructs are measurement-level distinct despite partial conceptual proximity in safety-related considerations.
